# Anti-Clutter Gaussian Inverse Wishart PHD Filter for Extended Target Tracking

**DOI:** 10.3390/s19235140

**Published:** 2019-11-23

**Authors:** Yuan Huang, Liping Wang, Xueying Wang, Wei An

**Affiliations:** College of Electronic Science, National University of Defense Technology, Changsha 410073, China; huangyuan09@nudt.edu.cn (Y.H.); wangliping17@nudt.edu.cn (L.W.); anwei@nudt.edu.cn (W.A.)

**Keywords:** extended target, target tracking, PHD filter, high clutter density

## Abstract

The extended target Gaussian inverse Wishart probability hypothesis density (ET-GIW-PHD) filter overestimates the number of targets under high clutter density. The reason for this is that the source of measurements cannot be determined correctly if only the number of measurements is used. To address this problem, we proposed an anti-clutter filter with hypothesis testing, we take into account the number of measurements in cells, the target state and spatial distribution of clutter to decide whether the measurements in cell are clutter. Specifically, the hypothesis testing method is adopted to determine the origination of the measurements. Then, the likelihood functions of targets and clutter are deduced based on the information mentioned above, resulting in the likelihood ratio test statistic. Next, the likelihood ratio test statistic is proved to be subject to a chi-square distribution and a threshold corresponding to the confidence coefficient is introduced and the measurements below this threshold are considered as clutter. Then the correction step of ET-GIW-PHD is revised based on hypothesis testing results. Extensive experiments have demonstrated the significant performance improvement of our proposed method.

## 1. Introduction

Extended target tracking (ETT) draws lots of attention in recent years because of its wide range of applications in traffic control [1], autonomous driving [2,3,4], person tracking [5,6] and etc. [7,8,9,10,11]. Since one extended target generates more than one measurement per time step, its shape information can be obtained. Using this information, the kinematic state and extent of the target can be estimated simultaneously. The extent of the target including the size, shape, and orientation can be further used for target identification.

The difference between point target tracking and extended target tracking lies in the measurement model and hypotheses. Point target generates at most one measurement per time step, while the extended target generates multiple measurements. Many algorithms were proposed to track point target based on point target hypothesis, such as probability hypothesis density (PHD) filter [12] and cardinalized PHD (CPHD) filter [13]. Since the extended target violates one measurement hypothesis, the number of targets will be overestimated if point target tracking algorithms are directly used for tracking extended targets. To address this problem, Mahler proposed an extended target tracking algorithm based on the inhomogeneous Poisson Point Process model (PPP model) [14] in random finite sets (RFSs) frame, namely extended target probability hypothesis density (ET-PHD) [15], Jiang et al. [16] proposed a novel time-matching ET-PHD filter, a Gaussian mixture implementation of the ET-PHD, called the extended target Gaussian inverse Wishart probability hypothesis density (ET-GIW-PHD) filter, has been presented in [17]. However, ET-PHD only estimates the kinematic state of the target (such as position, velocity) and does not estimate the extent of the target. Therefore, this method cannot extract the shape of the target. Nevertheless, the estimation of the target extent is important because it can be used to classify target and improve tracking accuracy [18,19,20].

Measurement partition is an important step in ETT. In ETT, measurements are partitioned into several non-empty subsets, each subset contains measurements that are all from the same source, either a single target or a clutter source, the subset is defined as cell. In ETT, the increase of measurements gives rise to the quick increase of the set partitions, thus the partition algorithm should be designed to achieve tractable computational complexity. Distance partition [17] is the most widely used method. Modified Bayesian adaptive resonance theory (MB-ART) [21] can also achieve good performance. For more details about other partition algorithms, please see [22,23,24].

One of the most important works in extended target tracking is how to model the target extent. To address this problem, the stick model is used for bicycle and pedestrian tracking [25,26]. The object extension is represented by a symmetric positive definite (SPD) random matrix [27], namely a random matrix (RM) model. Feldmann et al. [28] adapted the RM model for the case when the sensor error cannot be ignored. Lan et al. [29] took into account time variation and distortion of target extension in RM frame. In order to handle irregular shapes, a random hypersurface model (RHM) is introduced in [30,31,32]. Gaussian Processes (GP) was used to represent the target shape and achieved good performance [33,34,35,36]. Since shape estimation is similar to curving fitting, Kaulbersch et al. [37] applied a curve fitting method for shape estimation. $\mathrm{Granstr}\ddot{o}m$ et al. [38] proposed an extension model for specific sensor. $\mathrm{Granstr}\ddot{o}m$ et al. [39] proposed extended target Gaussian inverse Wishart PHD (ET-GIW-PHD) filter to incorporate widely used RM model into PHD filter and approximate the estimated PHD with an unnormalized mixture of Gaussian inverse Wishart (GIW) distributions. Later, $\mathrm{Granstr}\ddot{o}m$ et al. [40] proposed extended target Gamma Gaussian inverse Wishart PHD (ET-GGIW-PHD) filter to estimate the measurement rate and target state simultaneously. The combination of several RM model was used to model nonelliptic targets in [41,42]. As mentioned in [39], more experiments that test ET-GIW-PHD filters are needed, e.g., for data that contains more clutter than typical laser data does, this provides the general motivation for this paper. We found that the number of targets will be overestimated which degrades the final performance when severe clutters are partitioned into one cell in ET-GIW-PHD. More analyses are presented in Section 3. In this paper, we proposed an anti-clutter ET-GIW-PHD filter for better cardinality estimation performance.

The main contributions of this paper are twofold. First, the reason why ET-GIW-PHD overestimates the number of targets is discussed detailedly, and the probability of the measurement generated by clutter against different scenario parameters is presented. Second, in order to deal with the cardinality overestimation in ET-GIW-PHD, we proposed an anti-clutter ET-GIW-PHD filter which revises the correction step of ET-GIW-PHD with hypothesis testing. Hypothesis testing is introduced to determine the source of measurements in the cell, hypothesis testing results are integrated into the correction step in ET-GIW-PHD. In order to deal with the source of measurements correctly, the essential differences between the measurements of targets and clutter should be recognized. Since the variation of target state over time follows certain rules (motion model and shape transition model), the state of targets could be predicted while clutter could not. Then, the likelihood functions of targets and clutter are deduced. The likelihood functions are built based on not only the number of measurements but also the target state and spatial distribution of clutter. Since the likelihood ratio test statistic is proved to be subject to chi-square distribution, a threshold corresponding to the confidence coefficient is introduced, this threshold is used to determine the source of measurements in the cell. It worth note that the perfect sensor resolution is advocated as a theoretical hypothesis in this paper. In reality, the results in the Section 5 will be affected by the limited sensor resolution. Future work will tackle the sensor’s limited resolution.

The rest of the paper is outlined as follows. Section 2 reviews the ET-GIW-PHD filter. Section 3 discusses the reason why ET-GIW-PHD overestimates the number of targets. Our anti-clutter ET-GIW-PHD is presented detailedly in Section 4. We conduct experiments in different simulation scenarios to demonstrate the effectiveness of our proposed approach in Section 5; Conclusion is drawn in Section 6.

## 2. ET-GIW-PHD Review

In ET-GIW-PHD, both predicted PHD and corrected PHD can be approximated as an unnormalized mixture of Gaussian inverse Wishart distributions. Let $\zeta_{k}=\left\{x_{k},X_{k}\right\}$ be the sufficient statistics of the GIW components at time which contains kinematical state $x_{k}$ and extension state $X_{k}$ which is mathematically described by a symmetric and positively definite (SPD) random matrix. The iterative formulae for $\xi_{k}$ are obtained in [39]. More implementation details, such as pruning and merging, can also be found in [39].

Prediction:

(1) $$D_{k+1|k}\left(\xi_{k+1}\right)=\int p_{s}\left(\xi_{k}\right)p_{k+1|k}\left(\xi_{k+1}|\xi_{k}\right)\times D_{k|k}\left(\xi_{k}\right)d\xi_{k}+D_{k+1}^{b}\left(\xi_{k+1}\right),$$

where $p_{s}\left(\cdot \right)$ is the probability of survival, $p_{k+1|k}\left(\xi_{k+1}|\xi_{k}\right)$ is the state transition density, $D_{k+1}^{b}\left(\cdot \right)$ is the birth PHD, new target spawning is omitted [39].

Correction:

The corrected PHD $D_{k|k}\left(\xi_{k}\right)$ can be summarized as:

(2) $$D_{k|k}\left(\xi_{k}\right)=D_{k|k}^{ND}\left(\xi_{k}\right)+\sum_{p∠Z_{k}}\sum_{W\in p}D_{k|k}^{D}\left(\xi_{k},W\right),$$

where $p∠Z_{k}$ means that the measurement sets $Z_{k}$ are partitioned into non-empty cells, W∈p means that the cell *W* is in the partition p.

$D_{k|k}^{ND}\left(\xi_{k}\right)$ handles the undetected target case, because $D_{k+1|k}\left(\xi_{k+1}\right)$ is approximated as an unnormalized mixture of Gaussian inverse Wishart distributions, it is given by

(3) $$D_{k|k}^{ND}\left(\xi_{k}\right)=\sum_{j=1}^{J_{k|k-1}}w_{k|k}^{j}N\left(x_{k};m_{k|k}^{\left(j\right)},P_{k|k}^{\left(j\right)}⊗X_{k}\right)\mathrm{IW}\left(X_{k};v_{k|k}^{\left(j\right)},V_{k|k}^{\left(j\right)}\right),$$

where $J_{k|k-1}$ is the number of components of predicted PHD, $w_{k|k}^{\left(j\right)}$ is the weight of GIW component. N(x;m,P) means that a vector x is subject to Gaussian distribution with mean m and covariance P, IW(X;v,V) means that a matrix is subject to inverse Wishart distribution with degree of freedom *v* and inverse scale matrix V. ⊗ is the Kronecker product.

$D_{k|k}^{D}\left(\xi_{k},W\right)$ handles the detected target case, which is given by

(4) $$D_{k|k}^{D}\left(\xi_{k},W\right)=\sum_{j=1}^{J_{k|k-1}}w_{k|k}^{\left(j,W\right)}N\left(x_{k};m_{k|k}^{\left(j,W\right)},P_{k|k}^{\left(j,W\right)}⊗X_{k}\right)IW(X_{k};v_{k|k}^{\left(j,W\right)},V_{k|k}^{\left(j,W\right)}).$$

$w_{k|k}^{\left(j,W\right)}$ can be obtained by

(5) $$w_{k|k}^{\left(j,W\right)}=\frac{\omega_{p}}{d_{W}}e^{-\gamma^{\left(j\right)}}{\left(\frac{\gamma^{\left(j\right)}}{\lambda_{c}c_{k}}\right)}^{\left|W\right|}p_{D}^{\left(j\right)}\Lambda_{k}^{\left(j,W\right)}w_{k|k-1}^{\left(j\right)},$$

where

(6) $$d_{W}=\delta_{|W|,1}+\sum_{j=1}^{J_{k|k-1}}e^{-\gamma (j)}{\left(\frac{\gamma^{\left(j\right)}}{\lambda_{c}c_{k}}\right)}^{\left|W\right|}p_{D}^{\left(j\right)}\Lambda_{k}^{\left(j,W\right)}w_{k|k-1}^{\left(j\right)}$$

(7) $$\Lambda_{k}^{\left(j,W\right)}=\frac{1}{{\left(\pi^{\left|W\right|}|W|S_{k|k-1}^{\left(j,W\right)}\right)}^{\frac{d}{2}}}\frac{|V_{k|k-1}^{\left(j\right)}{|}^{\frac{v_{k|k-1}^{\left(j\right)}}{2}}\Gamma_{d}\left(\frac{v_{k|k}^{\left(j,W\right)}}{2}\right)}{|V_{k|k-1}^{\left(j,W\right)}{|}^{\frac{v_{k|k}^{\left(j,W\right)}}{2}}\Gamma_{d}\left(\frac{v_{k|k-1}^{\left(j\right)}}{2}\right)}$$

(8) $$\omega_{p}=\frac{\prod_{W\in P}d_{W}}{\sum_{P^{\prime }∠Z_{k}}\prod_{W^{\prime }\in P^{\prime }}d_{W^{\prime }}}.$$

$\Lambda_{k}^{\left(j,W\right)}$ presents the likelihood of the jth GIW component given the measurements of the Wth cell, $\omega_{p}$ is the weight of pth partition, $p_{D}^{\left(j\right)}$ is the detection probability of jth GIW component, $\gamma^{\left(j\right)}$ is the expected number of measurements generated by jth GIW component, $\lambda_{c}$ is the mean number of clutter measurements, $c_{k}$ is the spatial distribution of the clutter over the surveillance volume, $\delta_{i,j}$ is the Kronecker delta, |W| is the the number of measurements in the Wth cell, $S_{k|k-1}^{\left(j,W\right)}$ is innovation factor, $\Gamma_{d}\left(\cdot \right)$ is the multivariate Gamma function.

## 3. Analysis of ET-GIW-PHD

In ET-GIW-PHD, the calculation of $w_{k|k}^{\left(j,W\right)}$ is important. If the measurements in Wth cell are generated by clutter, $w_{k|k}^{\left(j,W\right)}$ is expected to be smaller than the pruning threshold, then the corresponding component will be eliminated and the clutter will be eliminated.

In Equation (5), $w_{k|k}^{\left(j,W\right)}$ contains two parts, one is the weight of the pth partition, denoted by $\omega_{p}$, the other is the weight of Wth cell in partition. Without loss of generality, only one partition is considered for clarity, therefore $\omega_{p}=1$. Substituting Equation (6) into Equation (5), we arrive at

(9) $$w_{k|k}^{\left(j,W\right)}=\frac{e^{-\gamma^{\left(j\right)}}{\left(\frac{\gamma^{\left(j\right)}}{\lambda_{c}c_{k}}\right)}^{\left|W\right|}p_{D}^{\left(j\right)}\Lambda_{k}^{\left(j,W\right)}w_{k|k-1}^{\left(j\right)}}{\delta_{|W|,1}+\sum_{l=1}^{J_{k|k-1}}e^{-\gamma (l)}{\left(\frac{\gamma^{\left(l\right)}}{\lambda_{c}c_{k}}\right)}^{\left|W\right|}p_{D}^{\left(l\right)}\Lambda_{k}^{\left(l,W\right)}w_{k|k-1}^{\left(l\right)}}.$$

From Equation (9), the numerator is a part of denominator, the measurements of Wth cell is used to correct each GIW component, then $\Lambda_{k}^{\left(j,W\right)}$ can be obtained, $w_{k|k}^{\left(j,W\right)}$ can be given based on some prior parameters, such as $p_{D}$, γ, $\lambda_{c}$ and $c_{k}$ (for brevity, the subscript and superscript are omitted here).

If the measurements in the cell are generated by clutter, the likelihood $\Lambda_{k}^{\left(j,W\right)}$ of each GIW component will be very small since clutter does not obey the kinematic and extent model of target. If the number of clutter measurements in the cell is equal to one, then |W|=1, $\delta_{|W|,1}=1$, $\sum_{l=1}^{J_{k|k-1}}e^{-\gamma (l)}{\left(\frac{\gamma^{\left(l\right)}}{\lambda_{c}c_{k}}\right)}^{\left|W\right|}p_{D}^{\left(l\right)}\Lambda_{k}^{\left(l,W\right)}w_{k|k-1}^{\left(l\right)}$ will be much smaller than 1 because the likelihood $\Lambda_{k}^{\left(j,W\right)}$ achieves a small value mentioned above and other parameters can be considered as constants, the value of $w_{k|k}^{\left(j,W\right)}$ will be close to 0 and is smaller than the pruning threshold, then the corresponding component will be eliminated and the clutter is eliminated. However, if the number of clutter measurements in the cell is more than one, then |W|≠1, $\delta_{|W|,1}=0$, Equation (9) is the normalization process. Although $\Lambda_{k}^{\left(j,W\right)}$ is close to zero, $w_{k|k}^{\left(j,W\right)}$ can still take a large value. In this case, ghost targets will emerge and the number of targets will be overestimated. Further details on numerical implementation can be found in Section 5.

According to the analysis above, if the measurement in the cell is clutter, $\sum_{l=1}^{J_{k|k-1}}e^{-\gamma (l)}{\left(\frac{\gamma^{\left(l\right)}}{\lambda_{c}c_{k}}\right)}^{\left|W\right|}p_{D}^{\left(l\right)}\Lambda_{k}^{\left(l,W\right)}w_{k|k-1}^{\left(l\right)}$ (denoted by $\sum_{l=1}^{J_{k|k-1}}\psi_{l,W}$) in Equation (9) should be added by 1. Otherwise, it should be added by 0 and the clutter can be eliminated. However, from Equation (9), if the cell contains only one measurement, $\sum_{l=1}^{J_{k|k-1}}\psi_{l,W}$ is added by 1, it means that the cell contains only one measurement is considered as clutter in ET-GIW-PHD. Otherwise, it is considered as a target if the cell contains more than one measurement. In fact, this assumption can be violated under strong clutter. The criterion whether measurements in the cell are generated by clutter based on only the number of measurements can be erroneous. A simple numerical calculation is shown below to illustrate this point.

In ET-GIW-PHD, the probability of the measurements of the cell generated by clutter is obtained based on the Bayesian theorem, see Equation (10). Note that, only the number of measurement is considered in this calculation.

(10) $$\begin{array}{cc} P(Z_{W}\subset C|n_{W}=1) & =1-P(Z_{W}\subset T|n_{W}=1)\\ \; & =\frac{P\left(n_{W}=1|Z_{W}\subset C\right)P\left(Z_{W}\subset C\right)}{P\left(n_{W}=1|Z_{W}\subset T\right)P\left(Z_{W}\subset T\right)+P\left(n_{W}=1|Z_{W}\subset C\right)P\left(Z_{W}\subset C\right)}, \end{array}$$

where $Z_{W}$ presents the measurements in cell, $n_{W}$ is the number of measurements in cell, C and T mean clutter and target respectively, $P(Z_{W}\subset C)$ and $P(Z_{W}\subset T)$ are the prior information.

The number of measurements generated by the target is subject to Poisson distribution with Poisson rate γ, the detection probability is $p_{d}$, then

(11) $$\begin{array}{cc} P(n_{W} & =1|Z_{w}\subset T)\;=\sum_{i=1}^{\infty }p_{d}{\left(1-p_{d}\right)}^{i-1}C_{i}^{1}\frac{\gamma^{i}}{i!}e^{-\gamma }\\ \; & \;\;\;\;\;\;\;\;\;\;\;\;\;\;\;\;\;\;\;\;\;\;\;=\sum_{j=0}^{\infty }p_{d}{\left(1-p_{d}\right)}^{j}\left(j+1\right)\frac{\gamma^{j+1}}{(j+1)!}e^{-\gamma }\\ \; & \;\;\;\;\;\;\;\;\;\;\;\;\;\;\;\;\;\;\;\;\;\;\;=p_{d}\gamma e^{-p_{d}\gamma }\sum_{j=0}^{\infty }\frac{{\left(\left(1-p_{d}\right)\gamma \right)}^{j}}{j!}e^{-(1-P_{d})\gamma }\\ \; & \;\;\;\;\;\;\;\;\;\;\;\;\;\;\;\;\;\;\;\;\;\;\;=p_{d}\gamma e^{-p_{d}\gamma }. \end{array}$$

where $C_{m}^{n}=\frac{n!}{m!(m-n)!}$ denotes the combinatorial number of the events that *m* out of *n*.

Remark: $p_{d}$ is not equal to $p_{D}$ in Equation (6). $p_{d}$ is the probability that one measurement generated by target or clutter is detected, while $p_{D}$ is the probability that an extended target will generate a measurement set [15]. $p_{D}$ can be derived if $p_{d}$ is already known.

The clutter measurements are assumed to be uniformly distributed over the surveillance area, and the number of clutter is subject to Poisson distribution with Poisson rate $\lambda_{c}$. So we have

(12) $$\begin{array}{cc} P(n_{W} & =1|Z_{w}\subset C)=\sum_{i=1}^{\infty }p_{d}{\left(1-p_{d}\right)}^{i-1}C_{i}^{1}\frac{{\lambda_{c}}^{i}}{i!}e^{-\lambda_{c}}\\ \; & \;\;\;\;\;\;\;\;\;\;\;\;\;\;\;\;\;\;\;\;\;\;=p_{d}\lambda_{c}e^{-p_{d}\lambda_{c}}. \end{array}$$

When the number of measurements is 1, the probability of the measurement in the cell generated by clutter is shown in Table 1 with different γ and $\lambda_{c}$. In this simulation, the prior information is set to 0.5, then $P\left(Z_{W}\subset C\right)=P\left(Z_{W}\subset T\right)=0.5$.

From Table 1 we can see that when γ=10, $\lambda_{c}=35$ and $p_{d}=0.9$, $P(Z_{W}\subset C|n_{W}=1)=0.013$. Although the cell contains only one measurement, the probability of the measurement in the cell generated by clutter is close to 0. Consequently, the criterion of ET-GIW-PHD does not work well in this case. when γ=10 and $\lambda_{c}=5$, $P(Z_{W}\subset C|n_{W}=1)=1$. In this case, the clutter is distinguished correctly based on the criterion of ET-GIW-PHD. In summary, the determination whether measurements are generated by clutter based on only the number of measurements can be erroneous.

## 4. Anti-Clutter ET-GIW-PHD

The difference between ET-GIW-PHD and anti-clutter ET-GIW-PHD is how to determine the source of measurements in the cell, specifically, the difference is how to obtain $d_{W}$ in Equation (6). Using only the number of measurements in ET-GIW-PHD to determine whether the measurements in the cell is the target or not may be erroneous. In contrast, our anti-clutter ET-GIW-PHD uses hypothesis testing to deal with this problem. The number of measurements, the kinematic state and extent state of target and clutter spatial distribution are taken into account to obtain the likelihood ratio test statistic.

There are two hypotheses:

(13) $$H_{0}:Z_{W}\subset C,$$

(14) $$H_{1}:Z_{W}\subset T,$$

where $Z_{W}=\left\{z_{1},z_{2},\ldots ,z_{n_{W}}\right\}$ is the measurements of Wth cell, $n_{W}$ is the number of the measurements, C and T represent clutter and target respectively.

The likelihood ratio test statistic for hypothese is given by

(15) $$\eta =\frac{L(Z_{W}|T)}{L(Z_{W}|C)},$$

where $L(Z_{W}|T)$ and $L(Z_{W}|C)$ are the likelihood to measure the set $Z_{W}$ given $Z_{W}\subset T$ and $Z_{W}\subset C$ respectively and $L(Z_{W}|T)$ and $L(Z_{W}|C)$ will be presented later.

If log(·) is applied to Equation (15), log(η) can be obtained.

(16) $$\log\eta =\log L\left(Z_{W}|T\right)-\log L\left(Z_{W}|C\right).$$

Because log(·) is monotony increase, log(η) is also the test statistic for hypothesis $H_{0}$ versus $H_{1}$. If these measurements are generated by the target, $L(Z_{W}|T)$ will achieve a large value and $L(Z_{W}|C)$ will be small. Consequently, the statistics log(η) will grow to a large value. Using a threshold, we can distinguish between targets and clutter. Specifically, if log(η) is greater than the threshold, the measurements in the cell is considered to be generated by targets. Otherwise, these measurements are considered to be clutter. The expression of $L(Z_{W}|T)$ and $L(Z_{W}|C)$ are given below, the setting of the threshold is discussed.

If the measurements are generated by a target, different extent models lead to a different expression of $L(Z_{W}|T)$. In this paper, $L(Z_{W}|T)$ is deduced based on the model in [27],

(17) $$\begin{array}{cc} L(Z_{W}|T) & =\sum_{j=n_{W}}^{\infty }p_{d}^{n_{W}}{\left(1-p_{d}\right)}^{j-n_{W}}C_{j}^{n_{W}}\frac{\gamma^{j}}{j!}e^{-\gamma }\cdot \prod_{i=1}^{n_{W}}N(z_{i};\left(H_{k}⊗I_{d}\right)x_{k},X_{k})\\ \; & =\sum_{j=n_{W}}^{\infty }p_{d}^{n_{W}}{\left(1-p_{d}\right)}^{j-n_{W}}\frac{j!}{n_{W}!\left(j-n_{W}\right)!}\frac{\gamma^{j}}{j!}e^{-\gamma }\cdot \prod_{i=1}^{n_{W}}N(z_{i};\left(H_{k}⊗I_{d}\right)x_{k},X_{k})\\ \; & =\frac{p_{d}^{n_{W}}\gamma^{n_{W}}}{n_{W}!}e^{-p_{d}\gamma }\sum_{j=n_{W}}^{\infty }\frac{{\left(\left(1-p_{d}\right)\gamma \right)}^{j-n_{W}}}{(j-n_{W})!}e^{-(1-p_{d})\gamma }\cdot \prod_{i=1}^{n_{W}}N(z_{i};\left(H_{k}⊗I_{d}\right)x_{k},X_{k})\\ \; & =\frac{p_{d}^{n_{W}}\gamma^{n_{W}}}{n_{W}!}e^{-p_{d}\gamma }\prod_{i=1}^{n_{W}}N(z_{i};\left(H_{k}⊗I_{d}\right)x_{k},X_{k}), \end{array}$$

where $I_{d}$ is an unit matrix with *d* dimension, $X_{k}$ is the extension of target at time *k*, $H_{k}$ is the 1D observation matrix.

The clutters are assumed to be uniformly distributed over the surveillance area [27], then

(18) $$\begin{array}{cc} L(Z_{W}|C) & =\beta_{FA}^{n_{W}}\sum_{j=n_{W}}^{\infty }\frac{\lambda_{c}^{j}}{j!}e^{-\lambda_{c}}C_{j}^{n_{W}}p_{d}^{n_{W}}{\left(1-p_{d}\right)}^{j-n_{W}}\\ \; & =\beta_{FA}^{n_{W}}\sum_{j=n_{W}}^{\infty }\frac{\lambda_{c}^{j}}{j!}e^{-\lambda_{c}}\frac{j!}{n_{W}!\left(j-n_{W}\right)!}p_{d}^{n_{W}}{\left(1-p_{d}\right)}^{j-n_{W}}\\ \; & =\beta_{FA}^{n_{W}}p_{d}^{n_{W}}\frac{\lambda^{n_{W}}}{n_{W}!}e^{-p_{d}\lambda_{c}}\sum_{j=n_{W}}^{\infty }\frac{{\left(\left(1-p_{d}\right)\lambda_{c}\right)}^{j-n_{W}}}{(j-n_{W})!}e^{-\left(1-p_{d}\right)\lambda_{c}}\\ \; & =\beta_{FA}^{n_{W}}p_{d}^{n_{W}}\frac{\lambda_{c}^{n_{W}}}{n_{W}!}e^{-p_{d}\lambda_{c}}\sum_{i=0}^{\infty }\frac{{\left(\left(1-p_{d}\right)\lambda_{c}\right)}^{i}}{i!}e^{-\left(1-p_{d}\right)\lambda_{c}}\\ \; & =\beta_{FA}^{n_{W}}\frac{p_{d}^{n_{W}}\lambda_{c}^{n_{W}}}{n_{W}!}e^{-p_{d}\lambda_{c}}, \end{array}$$

where $\beta_{FA}=\lambda_{c}c_{k}$, $\lambda_{c}$ is the mean number of clutter measurements, $c_{k}$ is the spatial distribution of the clutter over the surveillance volume.

Substitute Equations (17) and (18) into Equation (16), we have

(19) $$\begin{array}{cc} \log(\eta ) & =\log L\left(Z_{W}|T\right)-\log L\left(Z_{W}|C\right)\\ \; & =\sum_{j=1}^{n_{W}}\log(N\left(z_{j};\left(H_{k}⊗I_{d}\right)x_{k},X_{k}\right))+\log\left(\frac{p_{d}^{n_{W}}\gamma^{n_{W}}}{n_{W}!}e^{-p_{d}\gamma }\right)-\log\left(\beta_{FA}^{n_{W}}\frac{p_{d}^{n_{W}}\lambda_{c}^{n_{W}}}{n_{W}!}e^{-p_{d}\lambda_{c}}\right)\\ \; & =\sum_{j=1}^{n_{W}}\left\{-0.5\log2\pi -0.5\log|X_{k}|\right\}-\sum_{j=1}^{n_{W}}\left\{0.5\left(z_{j}-\left(H_{k}⊗I_{d}\right)x_{k}\right)X_{k}^{-1}{\left(z_{j}-\left(H_{k}⊗I_{d}\right)x_{k}\right)}^{T}\right\}\\ \; & \;\;\;\;+\left(-p_{d}\gamma +n_{W}\log p_{d}\gamma -\sum_{i=1}^{n_{W}}\log i\right)-\left(-p_{d}\lambda_{c}+n_{W}\log p_{d}\lambda_{c}-\sum_{i=1}^{n_{W}}\log i\right)-n_{W}\log\beta_{FA}\\ \; & =-0.5\sum_{j=1}^{n_{W}}\left\{\left(z_{j}-\left(H_{k}⊗I_{d}\right)x_{k}\right)X_{k}^{-1}{\left(z_{j}-\left(H_{k}⊗I_{d}\right)x_{k}\right)}^{T}\right\}-0.5n_{W}\log2\pi -0.5n_{W}\log\left|X_{k}\right|\\ \; & \;\;\;\;-p_{d}\left(\gamma -\lambda_{c}\right)+n_{W}\log\left(\frac{\gamma }{\lambda_{c}}\right)-n_{W}\log\beta_{FA}\\ \; & =-0.5G+D, \end{array}$$

where

(20) $$G=\sum_{j=1}^{n_{W}}\left(z_{j}-\left(H_{k}⊗I_{d}\right)x_{k}\right)X_{k}^{-1}{\left(z_{j}-\left(H_{k}⊗I_{d}\right)x_{k}\right)}^{T}$$

(21) $$\begin{array}{cc} D & =-0.5n_{W}\log2\pi -0.5n_{W}\log\left|X_{k}\right|-p_{d}\left(\gamma -\lambda_{c}\right)\\ \; & \;\;\;\;+n_{W}\log\left(\frac{\gamma }{\lambda_{c}}\right)-n_{W}\log\beta_{FA}. \end{array}$$

Because $z_{j}$ is subject to Gaussian distribution with mean $\left(H_{k}⊗I_{d}\right)x_{k}$ and covariance $X_{k}$, $z_{j}\propto N\left(\left(H_{k}⊗I_{d}\right)x_{k},X_{k}\right)$, thus

(22) $$G=\sum_{j=1}^{n_{W}}\left(z_{j}-\left(H_{k}⊗I_{d}\right)x_{k}\right)X_{k}^{-1}{\left(z_{j}-\left(H_{k}⊗I_{d}\right)x_{k}\right)}^{T}\propto X^{2}\left(n_{W}\right),$$

where *G* is subject to chi-square distribution with degree of freedom $n_{W}$. In Equation (21), γ, $\lambda_{c}$ and $\beta_{FA}$ are priori known, the volume of the target extension is proportional to $|X_{k}|$, the size of the target could be assumed to be unchanged, then *D* could be considered as a constant.

The confidence coefficient is set to α and a threshold is introduced (denoted by *g*), suppose hypothesi $H_{1}$ is true, then

(23) P(logη>g)=P(−0.5G+D>g)=P{G<2(D−g)}=α,

then

(24) $$2\left(D-g\right)=X_{1-\alpha }^{2}$$

(25) $$g=D-0.5X_{1-\alpha }^{2},$$

where

(26) $$\alpha =\int_{X_{\alpha }^{2}\left(n_{W}\right)}^{\infty }X^{2}\left(n_{W}\right)dx.$$

From Equation (23), the probability of log(η)<g is 1−α. Generally, α is set to be a value close to 1 and log(η)<g is a small probability event. If log(η)<g is satisfied, hypothesi $H_{1}$ should be rejected. Finally, we have

If log(η)<g, the measurements are generated by clutter, then

(27) $$d_{W}=1+\sum_{l=1}^{J_{k|k-1}}e^{-\gamma (l)}{\left(\frac{\gamma^{\left(l\right)}}{\beta_{FA}}\right)}^{\left|W\right|}p_{D}^{\left(l\right)}\Lambda_{k}^{\left(l,W\right)}w_{k|k-1}^{\left(l\right)}.$$

If logη≥g,the measurements are generated by targets, then

(28) $$d_{W}=\sum_{l=1}^{J_{k|k-1}}e^{-\gamma (l)}{\left(\frac{\gamma^{\left(l\right)}}{\beta_{FA}}\right)}^{\left|W\right|}p_{D}^{\left(l\right)}\Lambda_{k}^{\left(l,W\right)}w_{k|k-1}^{\left(l\right)}.$$

The pseudo-code for anti-clutter ET-GIW-PHD is illustrated in Table 2.

The difference between ET-GIW-PHD and anti-clutter ET-GIW-PHD lies in correction step. Pseudo-code for anti-clutter ET-GIW-PHD filter correction is shown in Table 3, pseudo-code for other steps (prediction, prune and merge etc) can be found in [39].

## 5. Simulation

In this section, the effectiveness of ET-GIW-PHD and anti-clutter ET-GIW-PHD were tested. Two scenarios with multiple targets were established. The surveillance area was set as [−1000m,1000m]×[−1000m,1000m], then $c_{k}$ is $2.5\times 10^{-7}$ under the assumption that clutter is uniformly distributed over the surveillance area. We set totally 100 time steps and the sampling time is 1 s.

In the first scenario, four targets moving along different lines were generated:

(29) $$\begin{array}{cc} x_{0}^{\left(1\right)} & =\left[-1000\;m,1000\;m,25\;m/s,-25\;m/s\right],t_{s}^{\left(1\right)}=5\;s,t_{e}^{\left(1\right)}=45\;s;\\ x_{0}^{\left(2\right)} & =\left[-1000\;m,-1000\;m,25\;m/s,25\;m/s\right],t_{s}^{\left(2\right)}=15\;s,t_{e}^{\left(2\right)}=55\;s;\\ x_{0}^{\left(3\right)} & =\left[1000\;m,-1000\;m,-25\;m/s,25\;m/s\right],t_{s}^{\left(3\right)}=25\;s,t_{e}^{\left(3\right)}=65\;s;\\ x_{0}^{\left(4\right)} & =\left[1000\;m,1000\;m,-25\;m/s,-25\;m/s\right],t_{s}^{\left(4\right)}=35\;s,t_{e}^{\left(4\right)}=75\;s; \end{array}$$

where $x_{0}^{\left(j\right)}$ is the initial state of jth target, $t_{s}^{\left(j\right)}$ is the born time of jth target, $t_{e}^{\left(j\right)}$ is the end time of jth target. The birth intensity in the first scenario is

(30) $$D_{b}\left(\xi \right)=\sum_{j=1}^{4}w_{b}N\left(x;x_{0}^{\left(j\right)},P_{b}⊗X_{k}\right)\mathrm{IW}\left(X_{k};v_{b},V_{b}\right),$$

where $w_{b}=0.03$, $P_{b}=\mathrm{diag}\left(\left[100,100\right]\right)$, $v_{b}=10$, $V_{b}=\mathrm{diag}\left(\left[100,100\right]\right)$.

**Figure 1:**
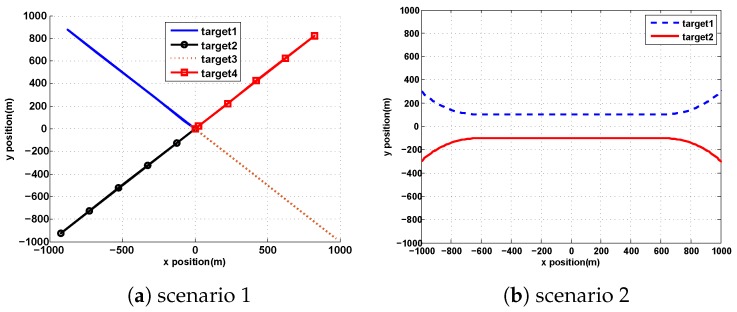
True trajectories of two scenarios: (**a**) four targets move along different lines in scenario 1. (**b**) Two targets move closeer gradually and then move in parallel before separating in scenario 2.

In the second scenario, two targets were born at (−1000 m, 300 m) and (−1000 m, −300 m), respectively at k=0 (*k* is time step). Next, they moved close gradually and then moved in parallel before separating. The birth intensity in the second scenario is

(31) $$D_{b}\left(\xi \right)=\sum_{j=1}^{2}w_{b}N\left(x;m_{j},P_{b}⊗X_{k}\right)\mathrm{IW}\left(X_{k};v_{b},V_{b}\right),$$

where $m_{1}=\left[-1000,300,25,-25\right]$, $m_{2}=\left[-1000,-300,25,25\right]$, $w_{b}=0.03$, $P_{b}=\mathrm{diag}\left(\left[100,100\right]\right)$, $v_{b}=10$, $V_{b}=\mathrm{diag}\left(\left[100,100\right]\right)$. The true trajectories of two scenario are shown in Figure 1.

The dynamic and measurement model are shown below. The target kinematic state is denoted as $x=[r_{x},r_{y},{\dot{r}}_{x},{\dot{r}}_{y}]$, where $r_{x}$ and ${\dot{r}}_{x}$ is the position and velocity in the *x* direction, likewise of *y* direction. The time evolution of kinematic state given by

(32) $$x_{k}^{\left(j\right)}=F_{k}^{\left(j\right)}x_{k-1}^{\left(j\right)}+w_{k}^{\left(j\right)},$$

where $x_{k}^{\left(j\right)}$ is the target state of jth target at time *k*, $w_{k}$ is the process noise of jth target and is the Gaussian white noise with zero mean and covariance $Q_{k}^{\left(j\right)}$, $F_{k}^{\left(j\right)}$ is the kinematic state transition matrix of jth target, given by

(33) $${F_{k}}^{\left(j\right)}=\left[\begin{array}{cccc} 1 & 0 & t & 0\\ 0 & 1 & 0 & t\\ 0 & 0 & 1 & 0\\ 0 & 0 & 0 & 1 \end{array}\right],{Q_{k}}^{\left(j\right)}=\Omega^{2}\left[\begin{array}{c} \frac{t^{2}}{2}\\ t \end{array}\right]\left[\begin{array}{cc} \frac{t^{2}}{2} & t \end{array}\right],$$

where *t* is the sampling time and Ω represents the acceleration error, t=1s and $\Omega =5\;m/s^{2}$ in this simulation.

In this simulation, the major and minor axes are 20 m and 15 m respectively for all targets. The major axis was aligned with the direction of motion of the target and the extent of these targets remained unchanged.

The measurement model can be expressed as

(34) $$z_{k}^{\left(j\right)}=\left(H_{k}⊗I_{d}\right)x_{k}^{\left(j\right)}+q_{k},$$

where $z_{k}^{\left(j\right)}$ is the measurements generated by the jth target at time *k*, $q_{k}$ is the measurement noise and is the Gaussian white noise with zero mean and covariance $R_{k}$, $H_{k}⊗I_{d}$ is the observation matrix, given by

(35) $$H_{k}⊗I_{d}=\left[\begin{array}{cccc} \begin{array}{c} 1\\ 0 \end{array} & \begin{array}{c} 0\\ 1 \end{array} & \begin{array}{c} 0\\ 0 \end{array} & \begin{array}{c} 0\\ 0 \end{array} \end{array}\right],R_{k}=\left[\begin{array}{cc} 1 & 0\\ 0 & 1 \end{array}\right].$$

where $H_{k}=\left[1,0\right]$, $I_{d}=\left[\begin{array}{cc} 1 & 0\\ 0 & 1 \end{array}\right]$.

In our experiment, the confidence coefficient α of anti-clutter ET-GIW-PHD is set to 0.99. A distance partition algorithm [17] is used for both filters, a measurement partition that contains several cells can be obtained for a given distance threshold. Clutter Poisson rate $\lambda_{c}$ is set to 35, then clutter density $\lambda_{c}c_{k}$ is $8.75\times 10^{-6}$ (the clutter density in this paper is higher than that of related references, such as [18,21]). The expected number of measurements generated by targets γ is set to 15. The probability of survival $p_{s}$ and the detection probability $p_{D}$ are assumed to be state independent and set to 0.99 and 0.98, respectively. The probability $p_{d}$ is set to 0.99.

Tracking results are evaluated using the optimal subpattern assignment metric (OSPA) [43], which is widely used to evaluate multiple-target tracking performance [39,40,41,42].

The OSPA distance is defined by

(36) $$d_{p}^{c}\left(\varkappa_{k},\hat{\varkappa_{k}}\right)={\left(\frac{1}{n}\left(\min_{\pi \in \Pi_{n}}\sum_{i=1}^{m}d^{c}{\left(x_{i},{\hat{x}}_{\pi (i)}\right)}^{p}+C^{p}\left(n-m\right)\right)\right)}^{1/p},$$

where m<n, $\varkappa_{k}=\left\{x_{k}^{\left(1\right)},x_{k}^{\left(2\right)},\ldots ,x_{k}^{\left(m\right)}\right\}$ is the true RFS at time k, $\hat{\varkappa }=\left\{{\hat{x}}_{k}^{\left(1\right)},{\hat{x}}_{k}^{\left(2\right)},\ldots ,{\hat{x}}_{k}^{\left(n\right)}\right\}$ is the estimated RFS, $\prod_{n}$ is the assignment results which assign ϰ to $\hat{\varkappa }$, *p* means p−norm, *c* is the penalty cost for cardinality mismatch. In this simulation, c=60 and p=2.

**Figure 2:**
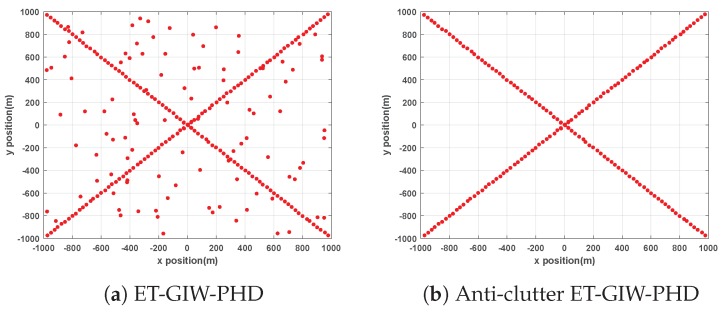
The obtained trajectories of ET-GIW-PHD and anti-clutter ET-GIW-PHD in scenario 1: (**a**) ET-GIW-PHD. (**b**) Anti-clutter ET-GIW-PHD.

**Figure 3:**
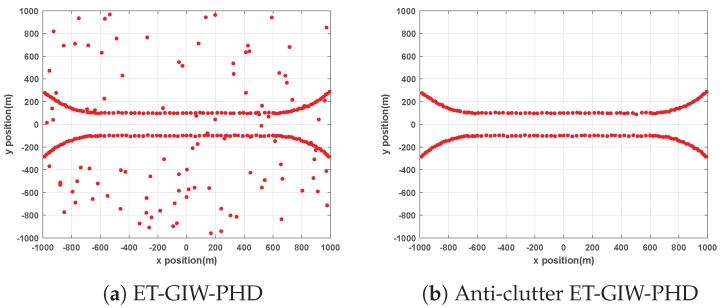
The obtained trajectories of ET-GIW-PHD and anti-clutter ET-GIW-PHD in scenario 2: (**a**) ET-GIW-PHD. (**b**) Anti-clutter ET-GIW-PHD.

ET-GIW-PHD and anti-clutter ET-GIW-PHD are applied to two scenarios mentioned above for performance evaluation. The trajectories generated by these two methods are presented in Figure 2 and Figure 3.

From Figure 2 and Figure 3 we can see that the trajectories of anti-clutter ET-GIW-PHD are almost identical to the true trajectories. Note that, in the results of ET-GIW-PHD, some peices of clutter are incorrectly considered as targets. However, our anti-clutter ET-GIW-PHD can deal with the clutter more correctly and achieves better performance.

**Figure 4:**
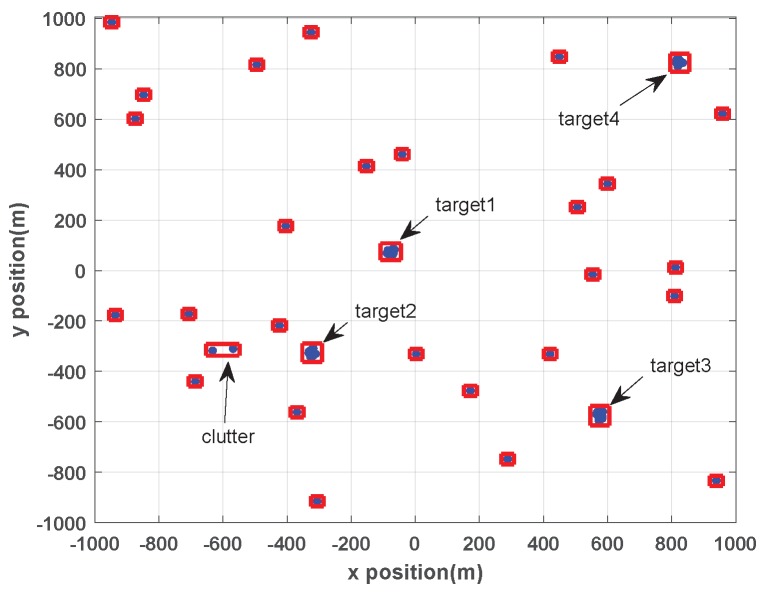
The partition result at k=40 in scenario 1.

To further verify the analysis in Section 3, the calculation of $w_{k|k}^{\left(j,W\right)}$ in Equation (9) at k=40 (*k* is time step) in scenario 1 is shown below. The partition result at k=40 is given firstly in Figure 4.

From Figure 4 we can see that the measurements of four targets are correctly clustered, and two clutter (marked with arrows in Figure 4) are incorrectly partitioned into one cell.

$e^{-\gamma^{\left(j\right)}}{\left(\frac{\gamma^{\left(j\right)}}{\beta_{FA,k}}\right)}^{\left|W\right|}p_{D}^{\left(j\right)}\Lambda_{k}^{\left(j,W\right)}w_{k|k-1}^{\left(j\right)}$ is denoted as $\psi_{j,W}$ for jth GIW component in the Wth cell, then

(37) $$w_{k|k}^{\left(j,W\right)}=\frac{\psi_{j,W}}{\delta_{|W|,1}+\sum_{l=1}^{J_{k|k-1}}\psi_{l,W}}.$$

From simulation results, the number of components of predicted PHD is 14 at k=40, then $J_{k|k-1}=14$, $\psi_{j,W}$ of the clutter cell (marked with arrows in Figure 4) is obtained and shown in Table 4.

The likelihood $\Lambda_{k}^{\left(j,W\right)}$ of each GIW component in this cell is very small since clutter does not obey the kinematic and extent model of target, therefore $\psi_{j,W}$ achieve small value as shown in Table 4.

Because the number of measurement in this cell is two, Equation (37) is represent as

(38) $$w_{k|k}^{\left(j,W\right)}=\frac{\psi_{j,W}}{\sum_{l=1}^{J_{k|k-1}}\psi_{l,W}}.$$

Equation (38) is a normalization process, $w_{k|k}^{\left(j,W\right)}$ of the clutter cell is shown in Table 5.

Although $\psi_{j,W}$ is small, $w_{k|k}^{\left(j,W\right)}$ may achieve a large value ($w_{k|k}^{\left(10,W\right)}=0.99$) and results in a ghost target. At k=40, the estimated number of targets was 5 while true number is 4. That is, the number of targets was overestimated.

**Figure 5:**
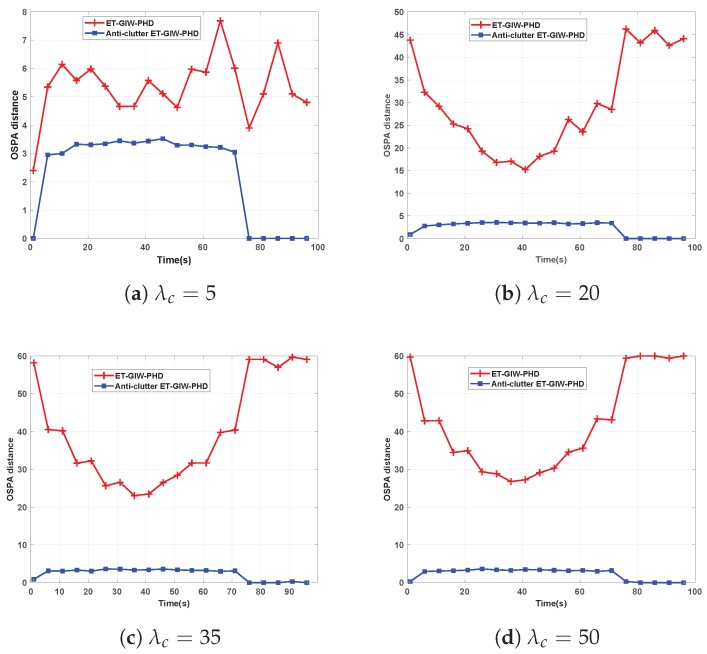
The optimal subpattern assignment metric (OSPA) distance of ET-GIW-PHD and anti-clutter ET-GIW-PHD under different Poisson rate of clutter in scenario 1: (**a**) Poisson rate $\lambda_{c}=5$. (**b**) Poisson rate $\lambda_{c}=20$. (**c**) Poisson rate $\lambda_{c}=35$. (**d**) Poisson rate $\lambda_{c}=50$.

**Figure 6:**
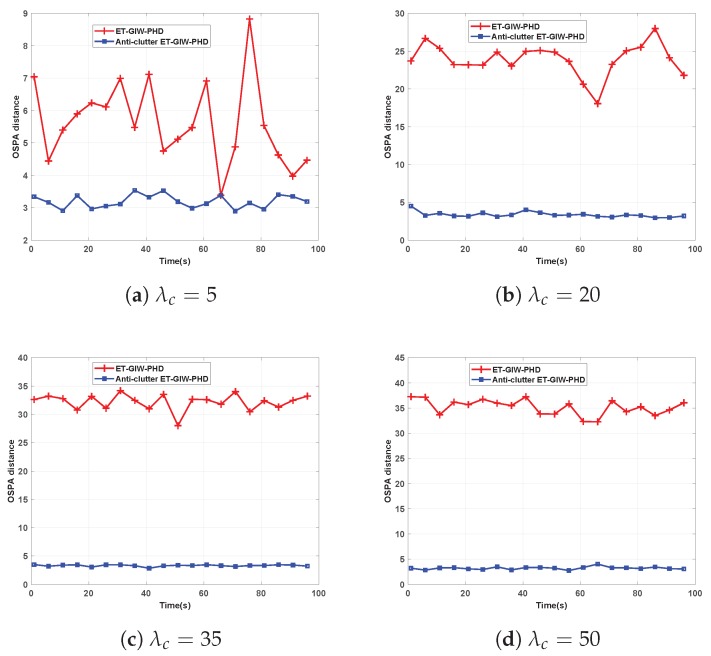
The OSPA distance of ET-GIW-PHD and anti-clutter ET-GIW-PHD under different Poisson rate of clutter in scenario 2: (**a**) Poisson rate $\lambda_{c}=5$. (**b**) Poisson rate $\lambda_{c}=20$. (**c**) Poisson rate $\lambda_{c}=35$. (**d**) Poisson rate $\lambda_{c}=50$.

To test the influence of the clutter density on tracking performance, ET-GIW-PHD and anti-clutter ET-GIW-PHD were tested under different numbers of clutter modeled as Poisson distribution with Poisson rate $\lambda_{c}$. The clutter measurements are assumed to be uniformly distributed over the surveillance area. The OSPA distance of these two filters under different Poisson rate $\lambda_{c}$ is shown in Figure 5 and Figure 6.

**Figure 7:**
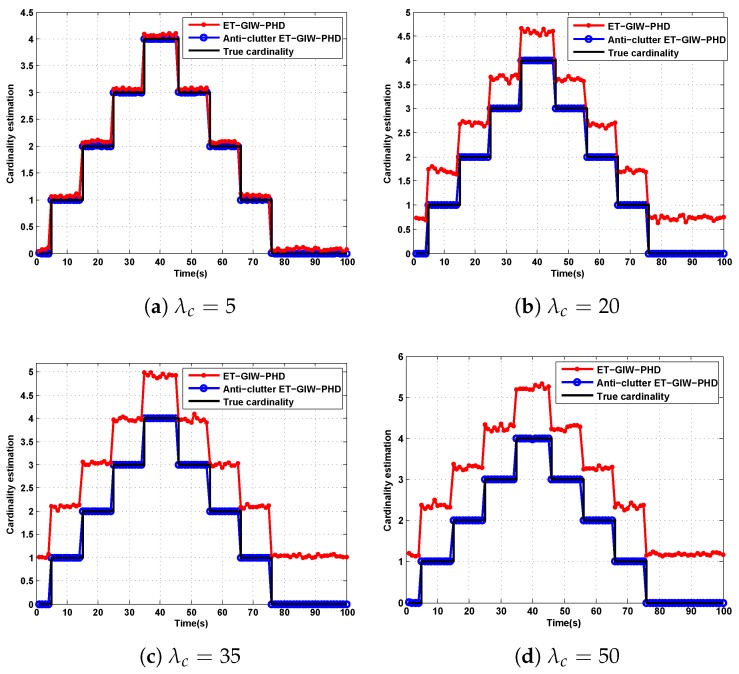
The cardinality estimation of ET-GIW-PHD and anti-clutter ET-GIW-PHD under different Poisson rate of clutter in scenario 1: (**a**) Poisson rate $\lambda_{c}=5$. (**b**) Poisson rate $\lambda_{c}=20$. (**c**) Poisson rate $\lambda_{c}=35$. (**d**) Poisson rate $\lambda_{c}=50$.

**Figure 8:**
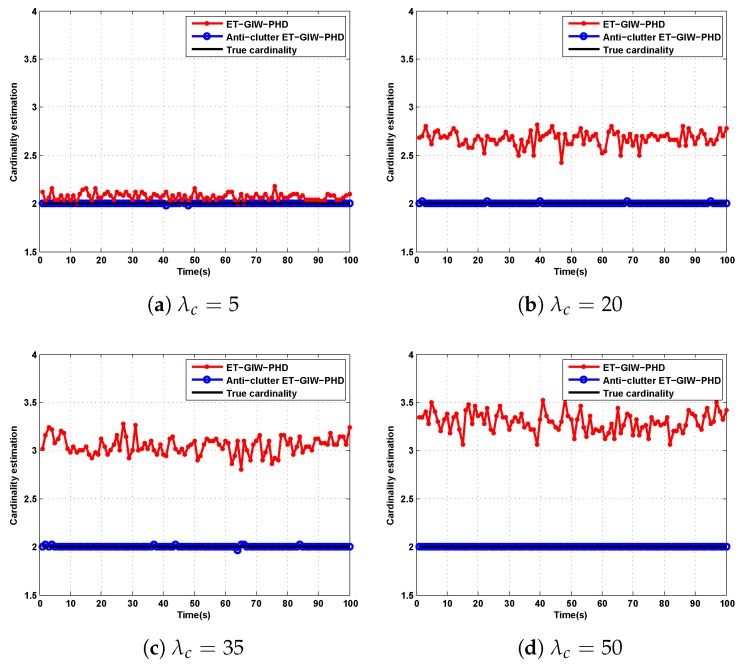
The cardinality estimation of ET-GIW-PHD and anti-clutter ET-GIW-PHD under different Poisson rate of clutter in scenario 2: (**a**) Poisson rate $\lambda_{c}=5$. (**b**) Poisson rate $\lambda_{c}=20$. (**c**) Poisson rate $\lambda_{c}=35$. (**d**) Poisson rate $\lambda_{c}=50$.

As we can see from Figure 5 and Figure 6, when $\lambda_{c}$ is small, ET-GIW-PHD achieves good performance. However, as $\lambda_{c}$ increases, the performance of ET-GIW-PHD degrades significantly. In contrast, our anti-clutter ET-GIW-PHD achieves superior performance with varying $\lambda_{c}$, which demonstrates that anti-clutter ET-GIW-PHD is more robust to clutter than ET-GIW-PHD. The results of cardinality estimation are shown in Figure 7 and Figure 8.

From Figure 7 and Figure 8 we can see that the cardinality estimation error of ET-GIW-PHD increases as the $\lambda_{c}$ grows. That is, ET-GIW-PHD cannot avoid the overestimation of cardinality under high clutter density. When clutter density is small, the clutter spreads apart. Thus, it is unlikely to partition more than one clutter into one cell. In the presence of severe clutter, the probability that multiple clutter being partitioned into one cell increases, and thus ET-GIW-PHD could overestimate the cardinality. However, our anti-clutter ET-GIW-PHD uses not only the number of measurement, but also target state and spatial distribution of clutter for better cardinality estimation performance. Using hypothesis testing, the measurements can be distinguished more correctly. Therefore, a better tracking performance can be achieved. Extensive experiments have demonstrated the effectiveness of anti-clutter ET-GIW-PHD.

## 6. Conclusions

In this paper, we propose an anti-clutter ET-GIW-PHD filter which revises the correction step of ET-GIW-PHD with hypothesis testing for better tracking performance under severe clutter. Our anti-clutter ET-GIW-PHD adopts a hypothesis testing method to distinguish between measurements from targets and clutter, hypothesis testing results are incorporated into the correction step. Specifically, likelihood functions are built to incorporate the number of measurements, the target state, and clutter spatial distribution in anti-clutter ET-GIW-PHD, the source of measurements in the cell is determined more correctly. Compared with ET-GIW-PHD, our method improves the cardinality estimation accuracy and achieves better tracking performance. The effectiveness of our method has been demonstrated by extensive experiments.

## Figures and Tables

**Table 1:** The probability of the measurement generated by clutter.

	$\lambda_{c}=5$	$\lambda_{c}=5$	$\lambda_{c}=25$	$\lambda_{c}=35$
	γ=5	γ=10	γ=10	γ=10
$p_{d}=0.6$	1	1	0.944	0.055
$p_{d}=0.7$	1	1	0.965	0.034
$p_{d}=0.8$	1	1	0.979	0.021
$p_{d}=0.9$	1	1	0.987	0.013

**Table 2:** Pseudo-code for anti-clutter extended target Gaussian inverse Wishart probability hypothesis density (ET-GIW-PHD) filter.

1: **Input:** Sequence of measurement sets
2: **Initialize:** parameter initialization
3: **for** k=1:K (*K* is totally time steps)
4: Measurements partition
5: Prediction
6: Correction, see Table 3.
7: Prune and merge
8: Extract target state
9: **end for**
10: **Output:** Sequence of estimated targets.

**Table 3:** Pseudo-code for anti-clutter ET-GIW-PHD filter correction.

1: **Input:** GIW components ${\left\{w_{k|k-1}^{j},\xi_{k|k-1}^{j}\right\}}_{j=1}^{J_{k|k-1}}$, measurements partitions ${\left\{p_{\rho }\right\}}_{\rho =1}^{n}$
2: **Undetected target case:**
3: **for** $j=1:J_{k|k-1}$
4: $w_{k|k}^{j}\leftarrow \left(1-\left(1-e^{-\gamma }\right)p_{D}\right)w_{k|k-1}^{j}\;\;\;\;\xi_{k|k}^{j}\leftarrow \xi_{k|k-1}^{j}$
5: **end for**
6: **Detected target case:**
7: l=0
8: **for** ρ=1:n
9: **for** $W=1:|p_{\rho }|$
10: l=l+1
11: **for** $j=1:J_{k|k-1}$
12: update $\xi_{k|k-1}^{j}$ using Kalman filter, see details in [39], $\xi_{k|k}^{j+l\cdot J_{k|k-1}}\overset{update}{\leftarrow }\xi_{k|k-1}^{j}$
13: $w_{k|k}^{\left(j+l\cdot J_{k|k-1},W\right)}\leftarrow e^{-\gamma^{\left(j\right)}}{\left(\frac{\gamma^{\left(j\right)}}{\lambda_{c}c_{k}}\right)}^{\left|W\right|}p_{D}^{\left(j\right)}\Lambda_{k}^{\left(j,W\right)}w_{k|k-1}^{\left(j\right)}$
14: $G^{j}=\sum_{j=1}^{n_{w}}\left(z_{j}-\left(H_{k}⊗I_{d}\right)x_{k|k-1}^{j}\right){\left(X_{k|k-1}^{j}\right)}^{-1}{\left(z_{j}-\left(H_{k}⊗I_{d}\right)x_{k|k-1}^{j}\right)}^{T}$
15: **end for**
16: $D=-0.5n_{W}\log2\pi -0.5n_{W}\log\left|X_{k|k-1}\right|-p_{d}\left(\gamma -\lambda_{c}\right)+n_{W}\log\left(\frac{\gamma }{\lambda_{c}}\right)-n_{W}\log\beta_{FA}.$
17: $\log\left(\eta \right)=-0.5\underset{j}{\arg\min}G^{j}+D$
18: $g=D-0.5X_{1-\alpha }^{2},$
19: $d_{W}^{\left(\rho ,W\right)}=\left\{\begin{array}{c} 1+\sum_{l=1}^{J_{k|k-1}}e^{-\gamma (l)}{\left(\frac{\gamma^{\left(l\right)}}{\beta_{FA,k}}\right)}^{\left|W\right|}p_{D}^{\left(l\right)}\Lambda_{k}^{\left(l,W\right)}w_{k|k-1}^{\left(l\right)}\;\;\;\log\eta <g\\ \sum_{l=1}^{J_{k|k-1}}e^{-\gamma (l)}{\left(\frac{\gamma^{\left(l\right)}}{\beta_{FA,k}}\right)}^{\left|W\right|}p_{D}^{\left(l\right)}\Lambda_{k}^{\left(l,W\right)}w_{k|k-1}^{\left(l\right)}\;\;\;\;\;\;\;\log\eta \geq g\; \end{array}\right.$
20: $w_{k|k}^{\left(j+l\cdot J_{k|k-1},W\right)}\leftarrow \frac{w_{k|k}^{\left(j+l\cdot J_{k|k-1},W\right)}}{d_{W}}$
21: **end for**
22: $\omega_{p_{\rho }}\leftarrow \Pi_{W=1}^{|p_{\rho }|}d_{W}^{\left(\rho ,W\right)}$
23: **end for**
24: $\omega_{p_{\rho }}\leftarrow \frac{\omega_{p_{\rho }}}{\sum_{\rho =1}^{n}\omega_{p_{\rho }}}$ for ρ=1:n
25: $J_{k|k}\leftarrow J_{k|k-1}\left(l+1\right)$ $J_{tmp}\;=\;J_{k|k-1}$
26: **for** ρ=1:n
27: **for** $j=1:J_{k|k-1}\left|p_{\rho }\right|$
28: $w_{k|k}^{\left(j+J_{tmp}\right)}\leftarrow w_{k|k}^{\left(j+J_{tmp}\right)}\omega_{p_{\rho }}$
29: **end for**
30: $J_{tmp}\leftarrow J_{tmp}+J_{k|k-1}\left|p_{\rho }\right|$
31: **end for**
32: **Output:** GIW components ${\left\{w_{k|k}^{j},\xi_{k|k}^{j}\right\}}_{j=1}^{J_{k|k}}$

**Table 4:** The $\psi_{j,W}$ of the clutter cell.

*j*	1	2	3	4	5	6	7
$\psi_{j,W}$	$4.7\times 10^{-21}$	$2.5\times 10^{-24}$	$1.4\times 10^{-19}$	$2.7\times 10^{-15}$	$2.9\times 10^{-10}$	$3.7\times 10^{-19}$	$3.5\times 10^{-70}$
*j*	8	9	10	11	12	13	14
$\psi_{j,W}$	$3.8\times 10^{-50}$	$4.4\times 10^{-57}$	$6.7\times 10^{-8}$	$2.6\times 10^{-30}$	$2.9\times 10^{-17}$	$4.7\times 10^{-14}$	$1.1\times 10^{-55}$

**Table 5:** $w_{k|k}^{\left(j,W\right)}$ of the clutter cell.

*j*	1	2	3	4	5	6	7
$w_{k|k}^{\left(j,W\right)}$	$6.9\times 10^{-14}$	$3.7\times 10^{-17}$	$2.1\times 10^{-12}$	$4.1\times 10^{-8}$	$4.3\times 10^{-3}$	$5.5\times 10^{-12}$	$5.1\times 10^{-63}$
*j*	8	9	10	11	12	13	14
$w_{k|k}^{\left(j,W\right)}$	$5.6\times 10^{-43}$	$6.5\times 10^{-50}$	0.99	$3.9\times 10^{-23}$	$4.3\times 10^{-10}$	$6.9\times 10^{-7}$	$1.7\times 10^{-48}$
